# Editorial: Novel technologies for sustainable and energy-efficient flow photochemistry

**DOI:** 10.3389/fchem.2023.1322556

**Published:** 2023-10-24

**Authors:** Michael Oelgemöller, Lijing Zhang, Fang Zhao, Yuanhai Su

**Affiliations:** ^1^ Faculty of Chemistry and Biology, Fresenius University of Applied Sciences, Idstein, Germany; ^2^ School of Chemistry, Dalian University of Technology, Dalian, China; ^3^ State Key Laboratory of Chemical Engineering, School of Chemical Engineering, East China University of Science and Technology, Shanghai, China; ^4^ Department of Chemical Engineering, School of Chemistry and Chemical Engineering, Shanghai Jiao Tong University, Shanghai, China

**Keywords:** photomicroreactors, flow photochemistry, photocatalysis, scale-up, intelligent flow synthesis

Due to the development of novel light-sources, methodologies and technologies, photochemistry has seen a remarkable renaissance in academia and industry (Baumann et al., 2014; Bonfield et al., 2020; Cohen et al., 2023). Many photochemical investigations are now routinely performed under continuous-flow conditions in purpose-designed reactors (Loubière et al., 2016; Buglioni et al., 2022). Successful examples of pre-industrial applications have subsequently been developed and realized (Basso and Capurro, 2021; Donnelly and Baumann, 2021; Zhang and Roth, 2023). Likewise, photocalytic materials can be easily incorporated into reactor channels, thus further advancing the potential of flow-photochemistry (Franchi and Amara, 2020; Thomson et al., 2020; Zuliani and Cova, 2021).

This Research Topic comprises of four submissions and highlights recent achievements in photochemical research. Li et al. developed a novel Fe^3+^-TiO_2_@CGS three-dimensional photoelectric system and applied it to the degradation of methylene blue. Under optimal operation conditions, the device reached a degradation yield of 99.98% after 60 min of photoelectrical treatment, clearly demonstrating the potential of this technology for the removal of organic contaminants. The constructed photoelectrical degradation reactor was equipped with inlet and outlet points, thus permitting (circulating) flow operation in future studies. Dinter et al. reported on the development of a flexible and affordable microfluidic photochemical flow reactor for rapid prototyping. The fabricated module was first utilized to optimize a photopinacolization reaction and was subsequently transferred to an application with DNA-tagged substrates. The study demonstrated the suitability of the developed modular flow photoreactor as a DNA-encoded library technology (DELT). Meinhardová et al. investigated the role of the lamp type for photocatalytic hydrogen production under batch and flow conditions. The authors initially established the efficiency of six commercial lamps in a batch reactor using a methanol-water solution and a NiO-TiO_2_ photocatalyst. Using a circulating microphotoreactors system incorporating TiO_2_ immobilized on borosilicate glass, continuous and reproducible hydrogen generation of 333.7 ± 21.1 µmol H_2_ or 252.8 ± 16.0 mmol·m^−2^ was achieved over a period of 168 h. Guo et al. summarized recent advances in catalyst development for the photocatalytic hydrogenation of nitrobenzene to aniline. In contrast to thermal methods, photocatalysis enables the sustainable production of the important platform chemical aniline at room temperature and low hydrogen pressures. Photocatalysts were divided into semiconductors, plasmonic metal-based catalysts and dyes, and the challenges, opportunities and future development prospects of these materials were described. Subsequent immobilization of these photocatalytic materials into flow devices may enable a continuous future production of aniline.

All contributions unambiguously demonstrate the potential and importance of flow-photochemistry and photocatalysis as sustainable and energy-efficient technologies.
